# Varied Practice in Laparoscopy Training: Beneficial Learning Stimulation or Cognitive Overload?

**DOI:** 10.3389/fpsyg.2016.00685

**Published:** 2016-05-10

**Authors:** Edward N. Spruit, Luca Kleijweg, Guido P. H. Band, Jaap F. Hamming

**Affiliations:** ^1^Cognitive Psychology, Institute of Psychology, Faculty of Social Sciences, Leiden UniversityLeiden, Netherlands; ^2^Department of Surgery, Leiden University Medical CenterLeiden, Netherlands; ^3^Leiden Institute for Brain and CognitionLeiden, Netherlands

**Keywords:** varied practice, laparoscopy training, motor skills, medical education, contextual interference

## Abstract

Determining the optimal design for surgical skills training is an ongoing research endeavor. In education literature, varied practice is listed as a positive intervention to improve acquisition of knowledge and motor skills. In the current study we tested the effectiveness of a varied practice intervention during laparoscopy training. Twenty-four trainees (control group) without prior experience received a 3 weeks laparoscopic skills training utilizing four basic and one advanced training task. Twenty-eight trainees (experimental group) received the same training with a random training task schedule, more frequent task switching and inverted viewing conditions on the four basic training tasks, but not the advanced task. Results showed inferior performance of the experimental group on the four basic laparoscopy tasks during training, at the end of training and at a 2 months retention session. We assume the inverted viewing conditions have led to the deterioration of learning in the experimental group because no significant differences were found between groups on the only task that had not been practiced under inverted viewing conditions; the advanced laparoscopic task. Potential moderating effects of inter-task similarity, task complexity, and trainee characteristics are discussed.

## Introduction

Acquisition and retention of complex perceptual and motor skills is often perceived as a challenge in various fields (aviation, driving, sports, medicine). With constant improvements in modern technology, more specialists require thorough and adequate training in order to operate new devices effectively. With increased popularity of minimally invasive procedures in health care, various medical professions require a new level of technical skills. For appendectomy and cholecystectomy, laparoscopy has become the default technique of operating. Minimally invasive procedures provide potential and proven benefits for patients (smaller scars, shortened hospitalization, faster return to daily activities), but are more difficult to learn for trainees. In laparoscopic surgery, practitioners need to attain perceptual and motor skills required to deal with the increase in complexity of the task at hand. Diminished tactile feedback, loss of depth perception cues, fewer degrees of motion freedom, amplified tremor, and counter-intuitive instrument movement (Gallagher and O’Sullivan, 2012) raise the inherent difficulty of the task.

Given these challenges, there has been an increase in demand for quality training of surgical residents before they start practicing in the operating room. In order to ensure patient safety, it is important that residents are prepared thoroughly and have a high retention and transfer of laparoscopy skills by the time they perform their first surgical procedure. There is a clear interest in both education and research to investigate best practices to conduct medical training (Gallagher and O’Sullivan, 2012; Spruit et al., 2013).

Variability of practice and frequent switching of different training tasks is a common suggestion in literature for efficient training (Schmidt and Bjork, 1992). However, it is thus far unclear whether variability of practice is beneficial in every context of training and to what extent the effect can be generalized (e.g., Hall et al., 1994; Brady, 2004; Barreiros et al., 2007; Jones and French, 2007). An important research question facing trainers is: How much variability is ideal during training of complex motor skills?

### The Contextual Interference Effect

The contextual interference (CI) effect (Battig, 1966; Shea and Morgan, 1979) is characterized by an impairment in performance during the skill acquisition phase of training, brought about by an increase of variability of training task parameters. When the training context changes frequently, the trainee experiences more interference of his motor actions, since different tasks require different actions. This finding is prevalent in the domain of memory (Schneider et al., 2002), but has also been widely tested in the field of motor skill acquisition (Magill and Hall, 1990). Examples of such variability in the motor domain are randomization (as opposed to block wise presentation) of the order of training tasks or frequently adjusting the conditions of a task (i.e., adjusting the distance to the target, the size of the ball or target in a basketball free-throw training task). Increasing the amount of CI tends to increase long-term retention of performance and flexibility of trained skills. Both are valuable for surgical residents since the interval between training and application on the job can be substantial and many different cases of anatomy can occur during surgical operations.

Two hypotheses are considered as an explanation for the effect (Boutin and Blandin, 2010). The elaboration hypothesis states that randomization of training tasks facilitate inter-task comparison during processing by the trainee, which leads to a more elaborate representation of different aspects of each training task. The reconstruction hypothesis suggests that due to the nature of more frequent task switching on a randomized training schedule, trainees are forced to rebuild the representation of their motor strategy whenever a different task is presented to them. In other words, they continually have to update their modus operandi whenever the training task changes, which strengthens the motor representation in long-term memory. It also helps trainees to stay engaged in learning.

The CI effect is similar to the spacing effect (Donovan and Radosevich, 1999), in that both center on the principle of increasing the effectiveness of learning episodes by splitting up training time into separate and different training intervals. When spacing training, this is achieved by utilizing multiple training sessions, whereas varied practice does so by creating more distinct training segments within one session. Both interventions force the trainee to update their working memory with instructions and task strategies more frequently than on a blocked or massed training schedule. An important difference is that spacing reduces the amount of retrograde interference (the extent to which a second training task practiced directly after a first training task compromises learning and retention of the first task, Shea and Graf, 1994; Brashers-Krug et al., 1996), whereas training with increased variability induces interference.

Both spacing and varied practice capitalize on the benefits of maximizing working memory recruitment throughout the learning process, which helps to keep trainees engaged in training. It is unclear whether this extra interference is beneficial or it becomes a heavy load on an inherently complex task, such as laparoscopy. Cognitive load theory (Paas et al., 2003) rests on the assumption that learning is optimal when trainees have enough spare cognitive resources available to reflect on what they are learning (germane load), next to the resources required to perform the task (intrinsic load) and the resources needed to comprehend instructions and filter noise (exogenous load). Hence, adding variable conditions to a complex task such as laparoscopy may lead to excess cognitive load (van Merriënboer et al., 2006) and result in a skill—challenge imbalance and a disruption of flow in the learning experience (Engeser and Rheinberg, 2008). Intrinsic germane load can be increased by adding more CI (more variability) to a practice schedule (Van Merriënboer and Sweller, 2010).

Brady (2004) found that the CI effect is more pronounced in basic experimental research, yielding moderate effect sizes, but small effect sizes are found for applied studies. A possible explanation is that most applied settings (like sports) already contain high variability even in blocked training conditions, whereas experimental lab settings with basic motor tasks are better able to control for variability of conditions. Also, basic laboratory research allows for rigorous control of variables, which have a more natural flow in applied settings (Barreiros et al., 2007). The current setting (laparoscopy simulator training) leaves us somewhere in the middle between a lab and applied setting.

The amount of research on varied practice and CI in medical training is limited, although it is a frequently suggested learning principle based on research in other fields (Schaverien, 2010; Spruit et al., 2013).

In the field of surgical training, Brydges et al. (2007) provided training on a bone-plating surgical task on three different practice schedules (whole-task training, blocked part-task training, and random part-task training) and found no support for the CI effect, since the whole-task training group showed more improvement than the random part-task training group. However, these results ought to be interpreted with caution since individual difference scores were used in order to account for group differences in baseline scores, rather than comparing post-test scores. Other research on laparoscopy training has shown that alternating the visual presentation during laparoscopy training increases the rate of automatization to the fulcrum effect (Jordan et al., 2000).

The aim of the current study is to investigate the effect of varied practice in laparoscopy training. Based on the CI literature, we first hypothesize that performance of the experimental (varied practice) condition will be impaired during and immediately following the acquisition phase of training, whereas performance will be enhanced at a retention session planned 2 months after training. Based on cognitive load theory, our second and competing hypothesis states that performance will be impaired in the experimental condition and the control condition will have superior performance on all sessions. We also expect all participants to improve in performance on all tasks from baseline to the end of training and retention, regardless of the condition.

## Materials and Methods

### Participants

Sixty medical and psychology students without prior experience in laparoscopy training were enrolled in the study (data in prior studies yielded no significant differences in laparoscopic performance and aptitude between medical and other university students, hence the inclusion of psychology students in the sample). Fifty-two students (39 female, 43 medical), all right-handed, age ranged from 18 to 29 years (mean = 21.54) completed all sessions. After completing the training, all participants received a certificate as a reward for participating in the study.

### Apparatus

Training centered primarily on practicing skills on a laparoscopic box trainer. Students practiced four basic and one advanced task with previously established construct validity (Kolkman et al., 2008). These tasks train perceptual and motor skills (instrument handling, depth perception, adjusting to the fulcrum effect) that are key to proficiency in laparoscopic surgery. The first task requires participants to stretch a rubber band around a set of 12 spikes. In this task a trainee learns to work with forces. In the second task, participants string a pipe cleaner through a set of four rings. This task aims to train bi-manual dexterity. The third task involves the placements of small beads on a pegboard and requires high precision of motor actions. In the fourth task, a circle should be cut in a rubber glove, which trains participants in exposure and dissection skills. In the advanced task, participants train the skill of intra-corporeal suturing. In the current study, participants were taught how to create three knots, starting with the needle in their right instrument, using two throws for the first knot (a throw is performed by moving the needle in a 360° motion around one of the instruments, creating a loop in the suture around the instrument). One throw was used for the second knot starting in the left instrument and one throw for the third knot starting from the right instrument (see the reference list for an online appendix with videos of all our laparoscopic training tasks^∗^^1^). To prepare participants for the advanced task, an open model of suturing was used in order to familiarize them with the procedure of knot tying before attempting the task in the box-trainer.

Performance of participants on the box-trainers was recorded on a connected PC with the use of a video splitter and grabster (Terratec Grabster AV 400/AV300 MX) to convert video output to separate video files via USB. The USB signal was converted to.mpg files by VLC Media Player for Windows.

Self-report questionnaires were used to acquire data on gender, age, dominant hand, academic year, prior sports, music, and gaming experience, goal orientation (Seijts and Latham, 2006) and growth mindset (Dweck, 2006). Goal orientation is a concept that differentiates between people who are more oriented toward learning versus being more oriented toward performance in their approach to goals. Growth mindset is a concept that refers to the extent that a person believes that skills and aptitude for learning is static (determined by innate and environmental factors) or dynamic (fluid, influenced by their own input and strategies). This data was collected to test comparability in these variables between the two conditions, since they may influence the learning curve.

### Training Programs

Participants were quasi-randomly assigned to the experimental (varied practice) condition (*n* = 28) or the control group (*n* = 24). Depending on their availability, participants were scheduled for three training sessions on a specific week day for three consecutive weeks. Both groups participated in three training sessions of 2 h per session. Each session consisted of 60 min of practice, 20 min of instructions, 10 min of break and a thirty minute period during which each participant’s performance was recorded (see **Table 1** for an overview of the training schedules). During the 1st week (training session I), participants practiced the four basic laparoscopic tasks and suturing on the open model in order to learn the basics of suturing and knot tying. In the 2nd week (training session II), intra-corporeal suturing was introduced and participants practiced all five laparoscopic tasks during the training sessions (II and III) in week two and three. Participants returned to the lab after 2 months for a retention session, during which performance was recorded without any prior practice on that session. Participants received standardized instructions from the trainers (first and second author) and a self-directed feedback (Strandbygaard et al., 2013) protocol was used: the majority of instructions were provided by video and feedback to trainees was minimized, and only provided if initiated by the trainees.

**Table 1:** Practice schedule of training session I for both conditions.

Blocked Condition		Contextual Interference (CI) Condition	
	
Activity	Minutes	Activity	Minutes
Welcome, planning, and informed consent	5	Welcome, planning, and informed consent	5
Instruction videos tasks 1–4	10	Instruction videos tasks 1–4	10
Recording of baseline tasks 1–4 and instruction on knot tying	30	Recording of baseline tasks 1–4 and instruction on knot tying	30
Practice task 1 (rubber band)	12	Practice round 1 (5 × 6 min on each of the 5 practice tasks)	30
Practice task 2 (pipe cleaner)	12		
Practice task 3 (placing beads)	12		
Break	10	Break	10
Practice task 4/5 (cutting circle/ knot tying)	12	Practice round 2 (5 × 6 min on each of the five practice tasks)	30
Practice task 4/5 (cutting circle/ knot tying)	12		
Debriefing	5	Debriefing	5


### Experimental and Control Condition

In the control condition, participants practiced each task in a set order, starting with practice on the easier tasks and progressing to the more difficult tasks. Each task was practiced for one time interval per session (see **Table 1**). The experimental group practiced each task for multiple smaller time intervals per session and switched more frequently between training tasks during each session than the control group. However, the total training time per task was equal for both conditions. Five box trainers with a training task were prepared and the trainees switched every six (training session one) or 3 min (training session two and three) between them when the experimenter indicated. The schedule for the varied practice group was determined randomly.

Also, participants in the experimental condition practiced each of the four basic laparoscopic tasks under inverted viewing conditions for half of the training time of these tasks (see **Table 2**). Inverted viewing conditions were achieved by flipping the laparoscopic box trainer by 180° across the front-back dimension and creating extra insertion points for the instruments on the other side of the box-trainer. This setup implied that the camera was now facing toward the trainee, which inverted the trainee’s perception of the movements made with the laparoscopic instruments. Under normal viewing conditions the camera was facing away, in line with the trainee’s field of vision. Each training task, trainees were unaware of which setup (inverted or regular viewing conditions) they were faced with until they started to practice. We chose this way of inverting perception (changing camera orientation) over other means (mirroring the screen monitor) because this setup is more congruent with a real surgical setting, where the angle of the laparoscope is frequently different from the angle of the surgeon’s field of view.

**Table 2:** Task order of the CI group training session 1.

Round 1	Round 2
Placing Beads	Pipe cleaner
Inverted rubber band	Inverted cutting circle
Inverted pipe cleaner	Rubber band
Cutting circle	Inverted placing beads
Knot tying	Knot tying


### Outcome Measures

The first and second author scored task completion times based on video recordings. Recordings at the beginning of the first session served to assess the baseline skill level. Performance was also measured at the end of the second and third training sessions. In order to test the durability of acquired skills, we assessed the participants’ retention 2 months after training, without any practice beforehand. In total, there were four moments of measurement.

### Statistical Analysis

Data distribution histograms were analyzed and Shapiro–Wilk tests were performed on the data to check for normality. We tested whether groups were comparable at baseline in terms of age, gender, dominant hand, academic year, academic specialization, musical, gaming, sports activity, and personality factors.

For each of the five training tasks, Mann–Whitney tests were done to test whether differences were present between groups at each stage of training (baseline, training session two, training session three, and retention). Wilcoxon signed-rank tests were done to check if improvements within trainees occurred between subsequent training sessions. We performed additional analyses (non-parametric correlations) to explore whether there were any interesting relations between questionnaire data and performance on the laparoscopic tasks.

Statistical significance was determined at *p* < 0.05, one-tailed for within-condition progress, two-tailed for between condition comparisons.

## Results

Fifty-two students (*N*_exp_ = 28, *N*_con_ = 24) completed all sessions and were included in data analysis. As predicted, data were non-normally distributed, so non-parametric tests were used.

If trainees were unable to complete a task within ten minutes, a score of 601 s (a score that would automatically be assigned as the highest rank in the non-parametric tests) was assigned. This was done to avoid selective drop-out from our sample based on poor performance in the cases where trainees were unable to complete a task. This was the case for 31 out of 988 moments of measurement.

### Baseline Check

Mann–Whitney tests revealed there were no differences between conditions in gaming and sports activity, although the experimental group (*Mdn* = 0/5, IQR = 0) practiced slightly less with musical instruments than the control group (*Mdn* = 0/5, IQR = 2), *U* = 247.5, *z* = -2.082, *p* = 0.041, *r*_rb_ = -0.29. The two conditions were similar in age (*Mdn*_exp_ = 21y; *Mdn*_con_ = 22y), *p* > 0.05. Independent samples *t*-tests showed comparable distributions on goal orientation and growth mindset among conditions.

Chi-square tests showed that the control group contained significantly more male participants (42%) than the experimental group (11%), *p* = 0.022. Also, the experimental group contained more psychology students (29%) compared to the control group (4%), *p* = 0.028. Male participants also performed better on most of the laparoscopic tasks (17 out of 19 measurements), but this difference was only significant in one instance (beads task retention, *p* = 0.01). We controlled for these factors after our main analysis.

On the first three basic laparoscopic tasks (elastic band, pipe cleaner, beads) there were no significant differences in baseline scores between conditions (see **Table 3**). For the circle cutting task, a significant trend in favor of the control group (*p* = 0.001) was found at baseline.

**Table 3:** Results of the Mann–Whitney tests with median completion times (in seconds) and effect sizes (*r*_rb_) for the five training tasks (horizontally).

	*Mdn*_exp_	*Mdn*_con_	*U*	*Z*	*p*	*r*_rb_
**Rubber band task**						
Baseline	146.0	0 133.0	320.0	-0.29	0.78	-0.04
Training session II	63.0^↓∗∗∗^	41.0^↓∗∗∗^	136.0	-3.67	<0.01	-0.51
Training session III	57.5^↓∗^	35.0^≈^	202.0	-2.46	0.01	-0.34
Retention	58.0^≈^	44.5^≈^	209.0	-2.33	0.02	-0.32
**Pipe cleaner task**						
Baseline	141.5	123.5	288.0	-0.88	0.38	-0.12
Training session II	63.0^↓∗∗∗^	42.0^↓∗∗∗^	143.5	-3.54	<0.01	-0.49
Training session III	54.0^≈^	43.5^≈^	198.5	-2.53	0.01	-0.35
Retention	66.0?^∗^	42.5^≈^	108.0	-4.19	<0.01	-0.58
**Beads task**						
Baseline	310.5	261.5	261.0	-1.38	0.17	-0.19
Training session II	172.5^↓∗∗∗^	121.5^↓∗∗∗^	167.5	-3.09	<0.01	-0.43
Training session III	147.5^≈^	119.5^≈^	240.0	-1.76	0.08	-0.24
Retention	158.5^≈^	126.5^≈^	204.0	-2.42	0.02	-0.34
**Cutting circle task**						
Baseline	428.0	303.5	166.0	-3.13	<0.01	-0.43
Training session II	183.5^↓∗∗∗^	130.0^↓∗∗∗^	224.5	-2.05	0.04	-0.28
Training session III	139.0^≈^	107.5^↓∗∗^	221.5	-2.10	0.04	-0.29
Retention	116.5^≈^	118.5 ^↑∗^	333.5	-0.05	0.97	-0.01
**Intra-corporeal suturing task**						
Training session II	386.5	359.0	333	-0.06	0.96	-0.01
Training session III	192.5^↓∗∗∗^	189.5^↓∗∗∗^	322	-0.26	0.80	-0.04
Retention	243.0^↑∗^	261.5^↑∗^	322.50	-0.25	0.81	-0.03


*Within-subject progress is indicated vertically for training session II, III, and the retention session compared to the previous session (^≈^for no significant difference; ^↓^/^↑^ for significant improvement/deterioration, ^∗^*p* < 0.05; ^∗∗^*p* < 0.01; ^∗∗∗^*p* < 0.001).*

### Within-group Progress across Training Sessions

The median scores at baseline, the end of training Sessions II, the end of training session III and at retention for the first four laparoscopic tasks are shown in **Table 3**. Participants in both groups significantly improved on all training tasks during training and showed deterioration from the end of training Session III to the retention session on the pipe cleaner task (experimental condition), cutting circle task (control condition) and the intra-corporeal suturing task (both conditions).

### Main Analysis: Between-condition Comparison

In **Table 3**, median scores of both conditions are compared for each task at each training session. Participants in the control condition performed significantly better at the basic tasks at Session II, Session III and retention, with the exception of beads (training session III) and circle cutting (retention). For the intra-corporeal suturing task, no significant differences between the conditions were found. Estimates of effect sizes show moderate effects for the first four basic laparoscopic tasks at Session II, Session III, and retention, but no effects for intra-corporeal suturing.

**FIGURE 1:**
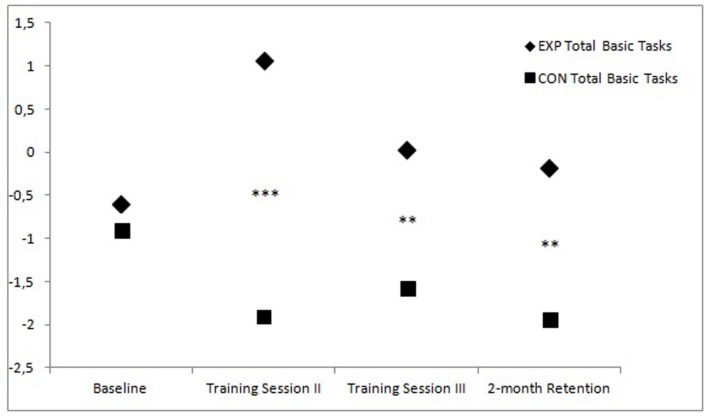
**Standardized total scores of the first four basic laparoscopic tasks at the start of the first training sessions (baseline), at the end of the second and third training session, and at retention for both training groups (Between-subject differences: ^∗^*p* < 0.05; ^∗∗^*p* < 0.01; ^∗∗∗^*p* < 0.001)**.

**FIGURE 2:**
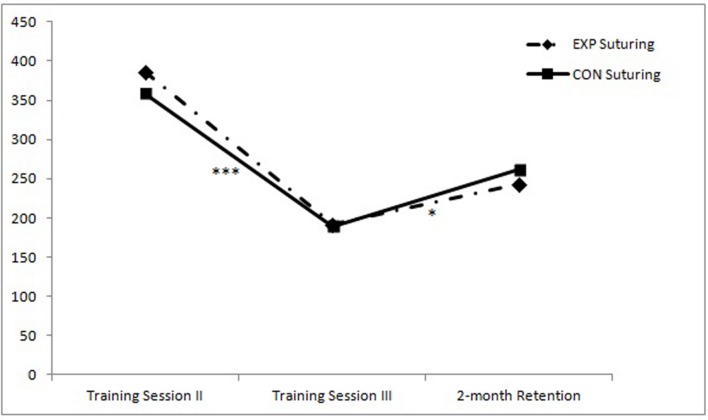
**Median completion times (in seconds) for the advanced laparoscopic task at the end of the second and third training session, and at retention for both training groups (Within-subject progress: ^∗^*p* < 0.05; ^∗∗^*p* < 0.01; ^∗∗∗^*p* < 0.001)**.

By standardizing and compounding the scores of the four basic laparoscopic tasks, this difference is clearly illustrated (see **Figure 1**). For the advanced laparoscopic task (suturing), the conditions have comparable scores (see **Figure 2**), although a trend can be observed where the median for the experimental condition is higher than the median of the control condition at the end of training Session II, roughly similar at the end of training Session III, but lower at retention.

### Extended Analysis and Confound Check

Given the lower scores for the control group at baseline on the circle task and a similar trend for the other basic tasks, we wanted to test whether these may have confounded our analysis. To achieve this, a *post hoc* case-controlled analysis was done for the standardized total baseline scores. We matched the two conditions in standardized total baseline scores by excluding the lowest scoring participants from the control condition and the highest scoring participants from the experimental condition until the two conditions had roughly equal mean rank scores in a Mann–Whitney test. The final sample for this analysis was 46 (*N*_exp_ = 24, *N*_con_ = 22). We found no significant differences relative to the results of our main analysis, although the observed trend for intra-corporeal suturing changed in favor of the experimental condition on all sessions (Training Session II: *Mdn*_exp_ = 386.5 → 303.0; *Mdn*_con_ = 359.0 → 393.5) (Training Session III: *Mdn*_exp_ = 192.5 → 179.5; *Mdn*_con_ = 189.5 → 199.5) (Retention Session: *Mdn*_exp_ = 243.0 → 220.5; *Mdn*_con_ = 261.5 → 261.5), but did not reach significance (*p* = 0.216; *p* = 0.386; *p* = 0.425; respectively). Matching groups for baseline scores on just the cutting circle task, musical activity, gender, or study did not substantially alter our results.

## Discussion

The results in the current study show a clear impairment of performance in the experimental group with varied practice on four basic laparoscopic tasks during training and at a retention session 2 months after training. Although the tempered performance on the short-term (session two and three) during training is characteristic of the CI effect (Brady, 2004), the experimental condition did not yield superior long-term performance (retention). Since retention performance is a better indicator for learning (Schmidt and Bjork, 1992), the additional CI in the experimental condition has likely impaired the learning process of the participants, which supports the second hypothesis (based on cognitive load theory).

What is notable, however, is that the scores on intra-corporeal suturing were not significantly different in the control and experimental group. Since this was the only task that was not practiced in inverted viewing conditions, it leads one to conclude that the inverted conditions have had a more detrimental effect as compared to the random training task schedule and the more frequent task switching. It is also possible that the random schedule had a positive effect, but it had been canceled out by the impaired learning caused by inverted viewing conditions. It is unclear whether or not the randomized schedule and frequent task switching has had a positive effect or not, but from the results of the intra-corporeal suturing task we can derive that it has not been a detrimental intervention.

### Inter-task Similarity and Contextual Interference

There are different ways of introducing CI and this allows for various ways in designing training. Switching between tasks that use a broader range of motor functions is known to lead to different types of CI (Magill and Hall, 1990). Switching between the pipe cleaner task and a rubber band task is not that big of a difference, since the tasks share a lot of similarities (same instruments, same perception, similar motor patterns). Different variations of the same type of motor task invoke the CI effect less frequently than switching between motor tasks that use different motor programs.

For instance, we could have had participants switch between a laparoscopic training task and a venipuncture procedure training task or a completely unrelated motor task (like a basketball throw) altogether. Switching between tasks that use different motor programs creates a more difficult learning setting, which facilitates CI (Magill and Hall, 1990). It is questionable whether such additional interference would have improved learning in the experimental condition in our current study given the high level of interference produced by the inverted viewing conditions. Nonetheless, it would be an interesting avenue for future studies and inter-task similarity is an important variable to consider when designing training of complex motor skills.

### Task Complexity

The tasks used in the current study are bound to a set of perceptual limitations. The notion that depth perception and tactile feedback are compromised makes for an inherently complex training task. Participants have to get accustomed to the fulcrum effect (Gallagher et al., 2008), the fact that whenever they move their hand in one direction, the tip of the corresponding instrument moves in the opposite direction. Task complexity was further increased for the experimental condition by adding the inverted viewing condition, where the fulcrum effect was canceled out. Switching between these (fulcrum effect/no fulcrum effect) conditions induced substantial CI during training.

In an earlier study, alternating between such conditions led to faster automatization to the fulcrum effect (Jordan et al., 2000). The criterion task in the mentioned study was not very complex (making incisions in an A4 paper) and only the horizontal axis was inverted by mirroring the monitor screen, rather than using the laparoscope from a different viewing angle. This results in just an inversion of left and right, as opposed to left and right and front and back, as was the case in the current study.

From our results, we conclude that the amount of CI may have led to a cognitive load that was too high for most of the participants. We suggest that the amount of CI has to be carefully gaged based on a trainee’s current proficiency level in order to attain the desired learning outcomes. The gaging principle also applies to the training context as stress can also have an impact on the amount of cognitive load a trainee can handle. This is especially relevant in fields such as aviation and medicine when simulating emergency scenarios (Taber, 2014).

### Trainee Characteristics

Novice trainees are impacted differently by CI than more experienced trainees (Brady, 2004). In the current study participants had no prior experience with laparoscopy training. During the learning process, the same training task becomes less challenging over time as the trainee practices and gains more skill. During training, the cognitive load it takes to execute a complex task remains stable over time (intrinsic load) and the surrounding conditions (quality of instructions, noise, task schedule) under which the task is performed remains relatively stable over time as well (exogenous load), but the cognitive load of learnable aspects of the task (germane load) diminishes as skill accumulates (Sweller et al., 1998). A novice without any prior experience on the task will be more taxed by germane load than the same novice at the end of a 3 weeks training. A plausible explanation for the current findings is that participants in the experimental condition received an increase in task complexity (inverse viewing conditions) and exogenous load (task switching) that was too high given the capacity of the cognitive load they were able to handle. Learning the laparoscopic tasks provided substantial germane load and the result of the varied practice intervention led to a training that became challenging to the extent of impairing, rather than facilitating learning.

In future designs, an option would be to test a gradual increase in CI, as is suggested by Porter and Magill (2010). This principle could be coupled with adaptive training, since no trainee’s skill level is the same when they initiate training and when they finish training. The amount of challenge is subjective to the trainee and task complexity and CI should be coupled with the skill of the trainee (Guadagnoli and Lee, 2004). Instead of burdening novices with too much CI, trainers can opt to introduce inverted viewing conditions to intermediate and advanced trainees that can handle the additional cognitive load. Testing this intervention at a later stage of training may allow trainees to accustom to the different angles of the laparoscope that they will likely face in the operating room.

### Study Limitations, Generalizations, and Conclusion

In the current study, participants were only measured on training tasks under regular viewing conditions. This may be seen as a limitation, since the control condition had more time-on-task practice during these circumstances as compared to the experimental condition. Measuring performance under inverted viewing conditions would alleviate this issue, but it is likely that this would be a serious challenge for the control condition, where this is a completely new context. Also, it is still unclear whether or not the trend observed for the suturing task was a positive CI effect of the frequent task switching or that it was due to sampling error. Future studies could investigate a two-by-two design with the inclusion and absence of the two training interventions (frequent task switching and alternating viewing conditions) to further clarify whether frequent task switching is truly beneficial for learning laparoscopic skills.

At first glance the current results may seem indicative of favoring the training design of the control condition, given their superior performance at the end of training and at retention. We emphasize, however, that task complexity needs to be taken into account when designing training. Also, low variability training conditions are typically associated with lower levels of transfer (van Merriënboer et al., 2006) and examples exist in the literature where higher CI conditions during simulation training lead to higher transfer when training less complex motor tasks (like tennis; Broadbent et al., 2015). Performance on a training simulator does not necessarily equal performance in the operating room (transfer). The laparoscope can be positioned in different angles throughout a laparoscopic procedure in the operating room and the contents of the human abdomen are more complex than a basic laparoscopic box-trainer. We encourage trainers to try to incorporate this principle in their training and research, so that less learning has to take place in the operating room and more learning can occur during training in the lab.

Based on our findings, we would urge trainers to be cautious initially when introducing new varied practice interventions to inexperienced trainees, but not to shy away from them either. Complex variations on training tasks and different camera orientations can be applied later in training, when trainees have attained a higher proficiency level. In this way, trainees are not overly challenged, but flexibility of their skills can still be enhanced.

## Author Contributions

ES. Primary investigator; study design; data collection; data analysis; writer first draft manuscript; corresponding author. LK. Study design; data collection; data analysis; feedback first manuscript. Dr. GB. Feedback study design; feedback data analysis; feedback; and rewriting of manuscript. Prof. JH. Feedback study design; feedback data analysis; feedback; and rewriting of manuscript.

## Conflict of Interest Statement

The authors declare that the research was conducted in the absence of any commercial or financial relationships that could be construed as a potential conflict of interest.

## Footnotes

1 ^∗^Online appendix containing videos of our laparoscopic training tasks and also serve as an illustration of our raw data: https://www.youtube.com/channel/UClH5gJxQ9lr9DXkUxtl7_9w
